# Tetrathiomolybdate Treatment Leads to the Suppression of Inflammatory Responses through the TRAF6/NFκB Pathway in LPS-Stimulated BV-2 Microglia

**DOI:** 10.3389/fnagi.2018.00009

**Published:** 2018-02-27

**Authors:** Zhuo Wang, Ya-Hong Zhang, Chuang Guo, Hui-Ling Gao, Man-Li Zhong, Ting-Ting Huang, Na-Na Liu, Rui-Fang Guo, Tian Lan, Wei Zhang, Zhan-You Wang, Pu Zhao

**Affiliations:** ^1^Department of Neurobiology, College of Life and Health Sciences, Northeastern University, Shenyang, China; ^2^Department of Hepatobiliary Surgery, General Hospital of Shenyang Military Area Command, Shenyang, China

**Keywords:** microglia, copper, tetrathiomolybdate, inflammation, ROS

## Abstract

Although the positive relationship between copper and Alzheimer's disease (AD) was reported by a lot of epidemiological data, the mechanism is not completely known. Copper is a redox metal and serves as a mediator of inflammation. Because the homeostasis of copper is altered in Aβ precursor protein (APP) and presenilin 1 (PS1) transgenic (Tg) mice, the using of copper chelators is a potential therapeutic strategy for AD. Here we report that a copper chelator, tetrathiomolybdate (TM), is a potential therapeutic drug of AD. We investigated whether TM treatment led to a decrease of pro-inflammatory cytokines *in vivo* and *in vitro*, and found that TM treatment reduced the expression of iNOS and TNF-α in APP/PS1 Tg mice through up-regulating superoxide dismutase 1 (SOD1) activity. *In vitro*, once stimulated, microglia secretes a variety of proinflammatory cytokines, so we utilized LPS-stimulated BV-2 cells as the inflammatory cell model to detect the anti-inflammatory effects of TM. Our results indicated that TM-pretreatment suppressed the ubiquitination of TRAF6 and the activation of NFκB without affecting the expression of TLR4 and Myd88 *in vitro*. By detecting the activity of SOD1 and the production of reactive oxygen species (ROS), we found that the anti-inflammatory effects of TM could be attributed to its ability to reduce the amount of intracellular bioavailable copper, and the production of ROS which is an activator of the TRAF6 auto-ubiquitination. Hence, our results revealed that TM-treatment could reduce the production of inflammatory cytokines by the suppression of ROS/TRAF6/AKT/NFκB signaling pathway.

## Introduction

Microglia, as principal immune cell in the central nervous system (CNS), serves as mediator of inflammation and degenerative diseases (Gonzalez-Scarano and Baltuch, 1999). The sustained activation of microglia is associated with several neurodegenerative diseases, such as multiple sclerosis and Alzheimer's disease (AD; Gonzalez-Scarano and Baltuch, 1999). It is reported that microglia can be activated by lipopolysaccharide (LPS), interferon (IFN)-γ, and β-amyloid (Aβ), promoting the production of nitric oxide (NO) and inflammatory cytokines such as, interleukin-1 beta (IL-1β) and tumor necrosis factor-alpha (TNF-α; Jana et al., 2007; Lee, 2013). These inflammatory cytokines contributed to several neurodegenerative diseases, including AD and Parkinson's disease (Liu and Hong, 2003; Block et al., 2007). Therefore, the inhibition of microglia activation can attenuate neuroinflammation, and may be useful for the treatment of inflammation-related neurodegenerative diseases (Perry et al., 2010; Kumar et al., 2012).

In the CNS, microglia is the major LPS-responsive cell. The receptor that responsible for LPS-induced microglia activation is the toll-like receptor 4 (TLR4). TLR4, also known as CD284, is a type I transmembrane protein, and its function is initiating innate immune responses. Lehnardt et al. reported that the neuronal death *in vitro* caused by the activation of TLR4 signaling pathway was depending on the presence of microglia, and LPS induced inflammation in wild-type mice could not be reappeared in TLR4 mutant mice (Lehnardt et al., 2003). Myeloid differentiation factor 88 (Myd88) is the adaptor protein of TLR4 that elicits activation of nuclear factor-κB (NFκB) to generate inflammatory cytokines (Wang et al., 2009; Li et al., 2011). Upon ligand stimulation, TLR recruits the adaptor proteins Myd88 and the IL-1 receptor associated kinase (IRAK)-1,4, which binds to tumor necrosis factor (TNF) receptor-associated factor 6 (TRAF6) to form complex to trigger the autopolyubiquitination of TRAF6, and subsequently triggers NF-κB activation (Wesche et al., 1997; Medzhitov et al., 1998; Deng et al., 2000; Martin and Wesche, 2002; Zhang, X. et al., 2013).

In addition to LPS, IFN-γ, and Aβ, the accumulation of redox-active transition metal ions, such as copper, iron, and zinc, is another risk factor of neurodegenerative diseases (Squitti et al., 2014). Our previous studies have shown that metal chelating compounds including trientine (Wang et al., 2013), clioquinol (Wang et al., 2012), and deferoxamine (Guo et al., 2013), protected neurons from the toxicity of metal-overloading. However, to date, the neuronal toxicity of copper in AD is still a controversial topic (Hureau and Faller, 2009; Hung et al., 2010; Schrag et al., 2011). The most well-confirmed finding was that copper was enriched in Aβ plaques. This led to the speculation that AD could be a copper-overloading disease. Recent research showed that copper exposure significantly elevated neuroinflammatory responses, resulting in the production of proinflammatory cytokines such as IL-1β and TNF-α (Kitazawa et al., 2016), and increased amyloid precursor protein (APP) accumulation to promote Aβ generation (Kitazawa et al., 2009). Recently, several *in vitro* studies showed that the early Aβ oligomer phase of its aggregation was more harmful, as a huge amount of reactive oxygen species (ROS) were produced in this stage (Tabner et al., 2001, 2010). Mayes et al. reported that copper [Cu(II)] bound with Aβ aggregates and then, promoted the shift between hydrogen peroxide (H_2_O_2_) and hydroxyl radical (·OH; Mayes et al., 2014). These free radical could promote inflammasome activation (Martinon, 2010). According to these studies, the spatial control or chelation of copper could be one of the choices to reduce the production of ROS in AD patients.

Tetrathiomolybdate (TM) is a copper chelator that was developed to treat Wilson's disease (Brewer et al., 2003). In the present study, we tested the therapeutic potential of TM in AD progression. It has been proved that TM inhibited vascular inflammation, adjuvant-induced arthritis and inflammation-associated cachexia (Omoto et al., 2005; Wei et al., 2012). However, as far as we are aware, there are no studies investigating the anti-inflammatroy effects of TM in microglia. The aim of the present study was to determine whether TM administration led to the suppression of inflammation in APP/PS1 Tg mice and in microglial cells via TLR4/Myd88/TRAF6/AKT/NFκB signaling pathway, and then function as a protector for neurodegenerative diseases.

## Materials and methods

### Reagents

See Supplemental Table 1 for the primary antibodies information. ELISA kits for detection of NO, IL-1β, and TNF-α were purchased from YanTai Science & Biotech and Beijing 4A Biotech Co. The DCFH-DA for the detection of ROS and Nuclear and Cytoplasmic Protein Extraction Kit were purchased from Beyotime Biotechnology China. Alexa Fluor 488-, Alex Fluor 594-conjugated secondary antibodies were purchased from Jackson ImmunoResearch, PA, USA. HRP-conjugated secondary antibodies were obtained from GE Healthcare Life Science, Beijing, China. The reagents for the quantitative real-time PCR (qPCR) were purchased from Promega Biotech Co., Ltd., Beijing, China. All primers used in qPCR were obtained from Sangon Biotech Co., Ltd., Shanghai, China. Tetrathiomolybdate (TM), 99.97% trace metals basis, was purchased from Sigma-Aldrich. Protein G beads were purchased from Thermo Fisher Scientific, China. SOD and GSH were purchased from Nanjing Jiancheng Bioengineering Institute, China.

### Mice and treatment

The APP/PS1 transgenic mice [B6C3-Tg (APPswe,PSEN1dE9)85Dbo/J(#004462)] and the wild-type mice were obtained from the Jackson Laboratory (Bar Harbor, ME, USA). All animal experimental procedures were approved by the Laboratory of Animal Ethical Committee of China Medical University. Six-month-old male mice were randomly divided into two groups (*N* = 6 per group): control group and TM group. In the control group, mice were given distilled water. In the TM group, mice were treated with TM (2.8 mg/kg/d) in drinking water (12 mg/L) daily for 3 months. Six-month-old wild-type mice were randomly divided into two groups (*N* = 6 per group): control group and TM group. In the control group, mice were given distilled water. In the TM-treated group, mice were treated with TM (2.8 mg/kg/d) in drinking water (12 mg/L) daily for 3 months.

### Tissue preparation

After 3 months treatment, the mice were sacrificed and the brains were removed quickly on ice. Half of the brains were frozen in −80°C for biochemical analysis. And the other half of brains were fixed into 4% paraformaldehyde.

### Cell culture

BV-2 cells were cultured in DMEM (high glucose) with 10% fetal bovine serum and L-glutamine in 5% CO_2_ at 37°C. Firstly, the cells pretreated with different doses of TM (6, 12, 24 μM) for 30 min (Wei et al., 2014), and then LPS was added and incubated with cells for 18 h. After 18 h of LPS treatment, the growth medium was collected for the detection of NO, IL-1β, and TNF-α (ELISA Kit) according to the manufacturer's instructions.

### Inductively coupled plasma mass spectrometry (ICP-MS)

To measure the content of copper in BV-2 cells, cells were pretreated with 6 μM TM for 30 min and then added with LPS to incubate for 18 h. After three times of PBS washing, the cells were collected and centrifuged at 800 rpm for 5 min. The supernatant was discarded, cells were digested with 500 μL of 90% HNO_3_ at 95°C for 30 min. The samples of cells were diluted, and then the content of copper in the diluted solutions was detected by using the 7500a- ICP-MS (Aglient Technologies InC., USA). Data acquisition mode (spectral analysis) is ^63^Cu.

### Quantitative real-time polymerase chain reaction (qPCR)

Total RNA was extracted from BV-2 cells and tissues using the Total RNA Kit (OMEGA Georgia, USA) according to the manufacturer's instruction and reverse transcribed into cDNA with the GoScript™ Reverse Transcription System (Promega, Madison, USA) on the Bio-rad CFX PCR System. The relative mRNA expression was calculated by Bio-Rad CFX software using ΔΔCt methods. All primers were span an exon-exon junction. All primers and the GenBank accession numbers were listed in Table 1. All the experiments were repeated at least for three times.

**Table 1:** Primers used for the quantitative real-time PCR.

**Primers**	**GenBank no**.	**Sequence**
TBP forward	NM_0136843	TGCACAGGAGCCAAGAGTGA
TBP reverse		AGCTGGGAAGCCCAACTTCT
IL-1β forward	NM_008361.4	AGCCAAGCTTCCTTGTGCAAGTGT
IL-1β reverse		GCTCTCATCAGGACAGCCCAGGT
TNF-α forward	NM_013693.3 NM_0012786011	AGCCCCCAGTCTGTATCCTT
TNF-α reverse		ACAGTCCAGGTCACTGTCCC
iNOS forward	NM_010927.4 NM_001313921.1 NM_001313922.1	AACAGAGCCCTCAGCAGCATCCAT
iNOS reverse		CCAGGTGTTCCCCAGGCAGGTAG
IL-6 forward	XM_021163844.1	TGTCTATACCACTTCACAAGTCGGAG
IL-6 reverse		GCACAACTCTTTTCTCATTTCCAC

### Immunofluorescence staining

BV-2 cells were treated with 6 μM TM for 30 min prior to LPS stimulation. After 18 h of LPS treatment, the cells were washed with PBS three times and then fixed with 4% paraformaldehyde for 15 min. And then were incubated with 0.1% Triton X-100 for 2 min after washing with PBS for three times. The sections were treated with 5% goat serum/PBS for 30 min and then incubated with primary antibodies overnight at 4°C. After washed, sections were incubated with Alex Fluor 594-conjugated secondary antibodies for 1.5 h. Images were taken using the Leica TCS SP8 laser scanning confocal microscope with 40 × objectives.

### Nuclear-cytosolic protein extraction

The extraction of the cytosolic and nuclear protein was performed according to the manufacturer's instructions kit. The concentration of the proteins was measured by Bradford assay (Ernst and Zor, 2010).

### Western blot

Cells and tissues were lysed with RIPA lysis buffer [50 mM Tris-HCL (pH 7.5), 150 mM NaCl, 1% NP-40, 1% sodium deoxycholate, 0.1% SDS, 5 mM EDTA, 25 mM NaF, and 2 mM Na_3_VO_4_, 1 mM PMSF] containing 1:100 diluted protease inhibitor cocktail (Sigma, St. Louis, MO, USA). The same amount of protein (50 μg) was separated on a 10% SDS-PAGE and transferred onto PVDF membranes, and probed with a panel of primary antibodies (Supplemental Table 1). All the experiments were repeated at least for three times.

### Immunoprecipitation

BV-2 cells were lysed with 1% NP40 lysis buffer [20 mM Tris-HCL (pH 7.5), 150 mM NaCl, 1% NP-40, 5 mM EDTA, 20 mM beta-glycerophosphate, 10% glycerol, 0.5 mM DTT, 25 mM NaF, 1 mM Na_3_VO_4_, 1 mM PMSF] containing 1:100 dilution protease inhibitor cocktail for 30 min on ice. Cell lysates were incubated with 2 μg TRAF6 antibody and protein G beads at 4°C overnight. Followed by washing, the proteins were eluted from beads using SDS sample buffer and analyzed by 10% SDS-PAGE.

### Statistical analysis

All data are expressed as the mean ± standard deviation (*SD*). Statistical analysis among two-group comparison was performed with 2-tailed Student's *t*-test with or without Welch's correction. Statistical analysis among three or more groups was performed with One-way ANOVA followed by Bonferroni's *post-hoc* test. *p* < 0.05 was considered to be statistically significant.

## Results

### TM reduced iNOS and TNF-α expression in the brain of APP/PS1 transgenic (Tg) mice

**Figure 1:**
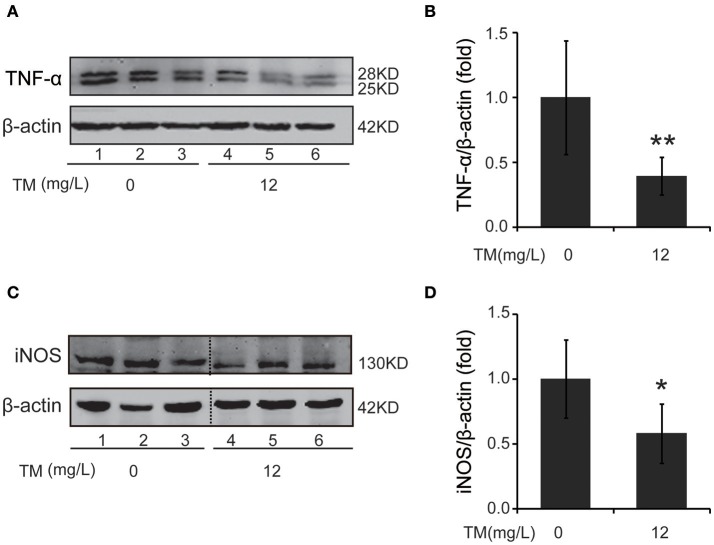
TM leads to suppression of pro-inflammatory cytokines in APP/PS1 Tg mouse brains. APP/PS1 Tg mice were pretreated with TM for 3 months and the brain homogenates were used to analysis the effects of TM on the production of pro-inflammatory cytokines. Lanes 1–3 represented different individuals of control APP/PS1 mice. Lanes 4–6 represented different individuals of TM-treated APP/PS1 mice. **(A,B)** Immunoblot images **(A)** and quantifications **(B)** show that TM reduced the expression of TNF-α. **(C,D)** Immunoblot images **(C)** and quantifications **(D)** show that TM reduced the expression of iNOS. Data are represented as means ± *SD. N* = 6 mice per group. ^*^p < 0.05, ^**^p < 0.01. The *p*-values were calculated using 2-tailed Student's *t*-test.

**Figure 2:**
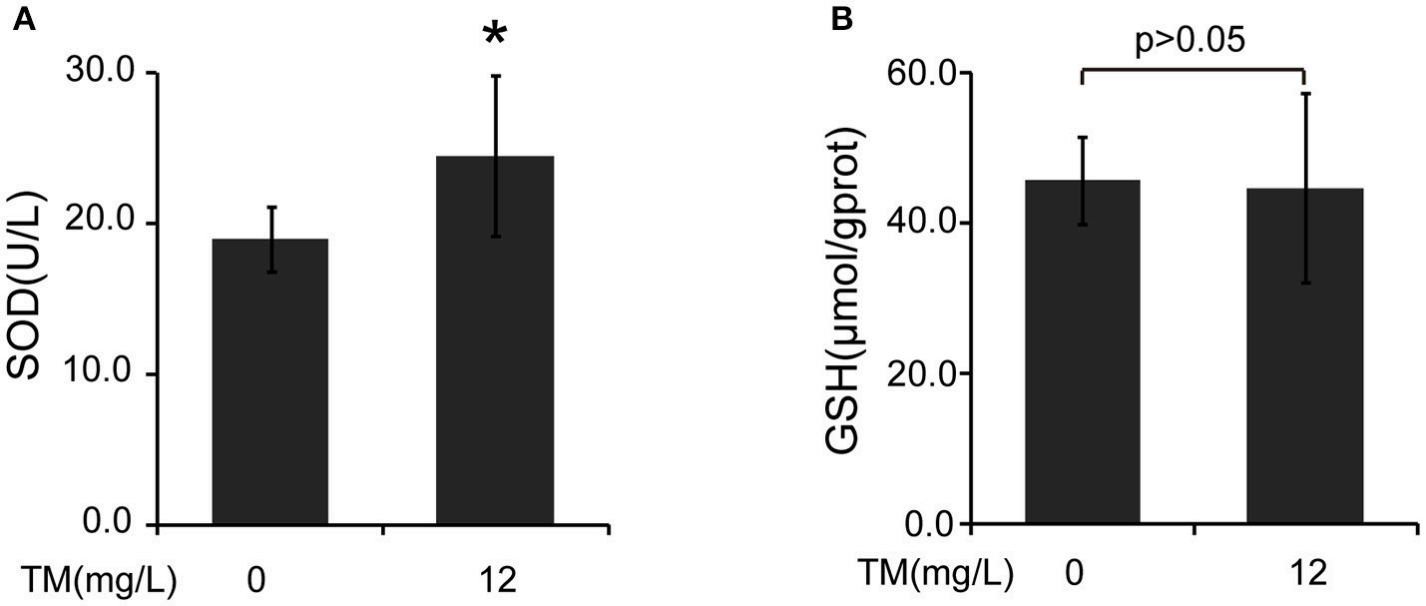
TM induces the activity of SOD in APP/PS1 Tg mouse brains. The brain homogenates were used to analysis the effects of TM. **(A)** TM increased the activity of SOD compared with control. **(B)** TM had no effect on the activity of GSH. Data are represented as means ± *SD. N* = 6 mice per group. ^*^p < 0.05. The *p-*values were calculated using 2-tailed Student's *t*-test.

Inflammatory cytokines, such as IL-1β and TNF-α, are believed to induce neurodegeneration, including AD (Liu and Hong, 2003). Therefore, we detected the expression of iNOS and inflammatory cytokines, such as IL-1β, IL-6, and TNF-α, in the brain of TM-treated APP/PS1 Tg mice. After being treated with TM for 3 months, although the mRNA expression of IL-1β and IL-6 were not reduced by TM-treatment (Supplementary Figure 1), the expression of TNF-α (Figures 1A,B) and iNOS (Figures 1C,D) were significantly reduced in TM-treated APP/PS1 Tg mice as compared with control mice. Meaning while, TM had no effect on the expression of iNOS and TNF-α in wild-type mice (Supplementary Figure 2). These findings suggested that TM could reduce inflammatory responses in the brain of APP/PS1 Tg mice. As a huge amount of ROS are produced among Aβ aggregation, the enzymatic and non-enzymatic antioxidant defenses including superoxide dismutase (SOD), glutathione peroxidase (GPX), catalase (CAT), ascorbic acid (vitamin C), α-tocopherol (vitamin E), glutathione (GSH), β-carotene, and vitamin A protect cells from oxidative-stress injury. In order to determine whether the anti-inflammatory effects of TM is depending on copper associated signaling pathways, we detected the SOD1 activity in TM-treated APP/PS1 mice and control mice. Our results indicated that TM increased the levels of SOD1 (Figure 2A), but had no effect on GSH (Figure 2B), suggesting that TM specifically reduced bioavailability of copper in APP/PS1 Tg mice (Figure 2).

### TM decreased inflammatory effects via reducing copper levels, iNOS expression and no secretion in LPS-induced microglial cells

**Figure 3:**
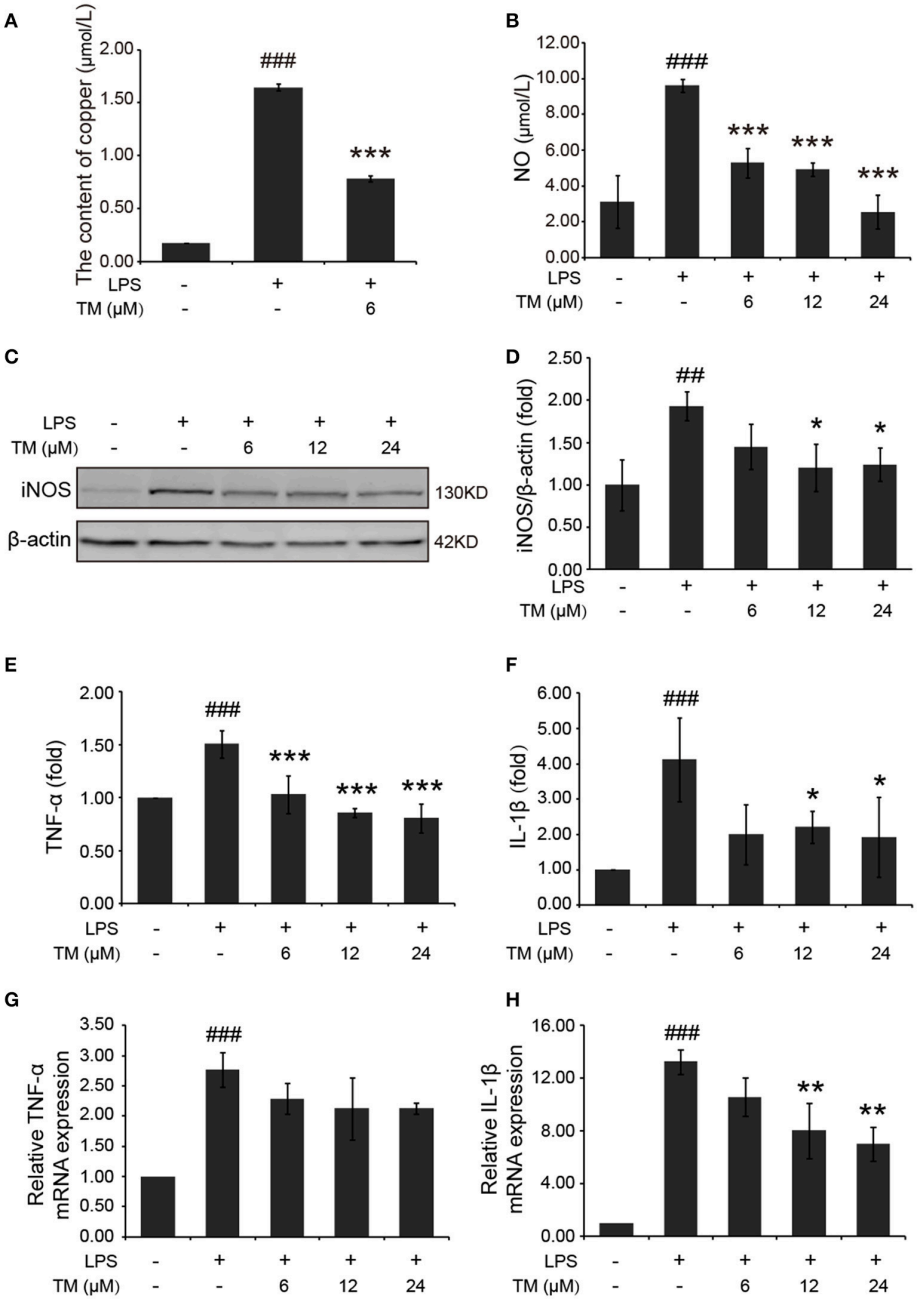
TM-pretreatment reduces the levels of copper and inhibits the production of NO and the expression of pro-inflammatory cytokines in LPS-induced BV-2 cells. BV-2 cells were pretreated with 6, 12, 24 μM of TM followed by treatment with 1 μg/ml LPS for 18 h. **(A)** The levels of copper were detected by using the ICP-MS. **(B)** NO production was detected by Griess agent. **(C,D)** Immunoblot images **(C)** and quantifications **(D)** show that TM-pretreatment suppressed iNOS expression in LPS-induced BV2 cells. **(E,F)** ELISA assay data show that TM-pretreatment decreased the release of TNF-α and IL-1β in LPS-induced BV2 cells. The absolute values ranges of TNF-α and IL-1β were 1000–2000 pg/ml and 102–993 pg/ml. **(G,H)** Quantitative real-time PCR (qPCR) data show that TM-pretreatment blocked TNF-α and IL-1β mRNA expression in LPS-induced BV2 cells. Data are represented as means ± *SD*. of at least three independent experiments (*N* ≥ 3). ^***^p < 0.001 ^**^p < 0.01, ^*^p < 0.05 compared with the LPS; ###*p* < 0.001, ##*p* < 0.01 compared with the control. The *p-*values were calculated by One-way ANOVA followed by Bonferroni's *post-hoc* test.

Inflammatory cytokines are predominantly produced by microglia in the brain. Then we determined whether TM inhibited the secretion of inflammatory cytokines in microglial cells. Firstly, we detected the effect of TM on the content of copper. The results showed that copper levels were increased in LPS-induced group, as compared with the control group, and the increased copper in LPS-induced BV-2 cells could be abolished by TM-pretreatment (Figure 3A). As a hippocampal neurotransmitter and a neurotoxic mediator, NO production is positively associated with the impairment of serotonergic transmission in the brain under stressful situations (Joca et al., 2007), and its neurotoxicity has been attributed to the inhibition of mitochondrial respiration (Bolanos et al., 1997). To evaluate the anti-inflammatory responses of TM on the microglial cells, the NO secretion and the iNOS expression in LPS-induced microglial cells were detected in mouse microglial cells (BV-2 cells). After being pretreated with TM (6, 12, 24 μM) for 30 min followed by stimulation with LPS (1 μg/mL) for 18 h, the NO production of BV-2 cells was detected by using Griess agent. The results showed that NO secretion was increased in the LPS-induced group, as compared with the control group (Figure 3B *p* < 0.001), and the increased-NO secretion in LPS-induced BV-2 cells can be abolished by TM-pretreatment (Figure 3B, *p* < 0.001). To further understand the mechanism by which TM treatment reduced NO production, we examined iNOS mRNA and protein levels by RT-PCR and western blot, respectively. As showed in Supplementary Figure 3 and Figures 3C,D, the expression of iNOS mRNA (*p* < 0.001) and protein (*p* < 0.01) were significantly upregulated in the LPS-induced group, as compared with the control group, and the upregulated expression of iNOS mRNA and protein could be abolished by different dose of TM-pretreatment (Supplementary Figure 3 and Figures 3C,D, *p* < 0.05), but the intrigue thing was that no dose-dependent effect was observed in this experiment. Taken together, the copper chelator TM decreased inflammatory effects via reducing iNOS expression and NO secretion in LPS-induced microglial cells.

### TM inhibited the production of pro-inflammatory cytokines induced by LPS

In addition to NO secretion, LPS stimulation also increased the production of inflammatory cytokines, such as TNF-α and IL-1β (Liu et al., 2011). Then we detected the effect of TM on the production of TNF-α and IL-1β in LPS-induced BV-2 cells. The levels of TNF-α and IL-1β in the growth media were detected by ELISA. Consistent with previous studies, our results indicated that LPS stimulation significantly increased the release (Figures 3E,F) and the mRNA expression (Figures 3G,H) of TNF-α (Figures 3E,G) and IL-1β (Figures 3F,H) in BV-2 cells (*p* < 0.001), and the increased TNF-α and IL-1β can be suppressed by different dose (6, 12, 24 μM) of TM-pretreatment (Figures 3E-H), suggesting that TM decreased the production of IL-1β and the release of TNF-α.

### TM decreased the production of inflammatory cytokines via TRAF6/AKT/NFκB signaling pathway by decreasing the production of ROS

**Figure 4:**
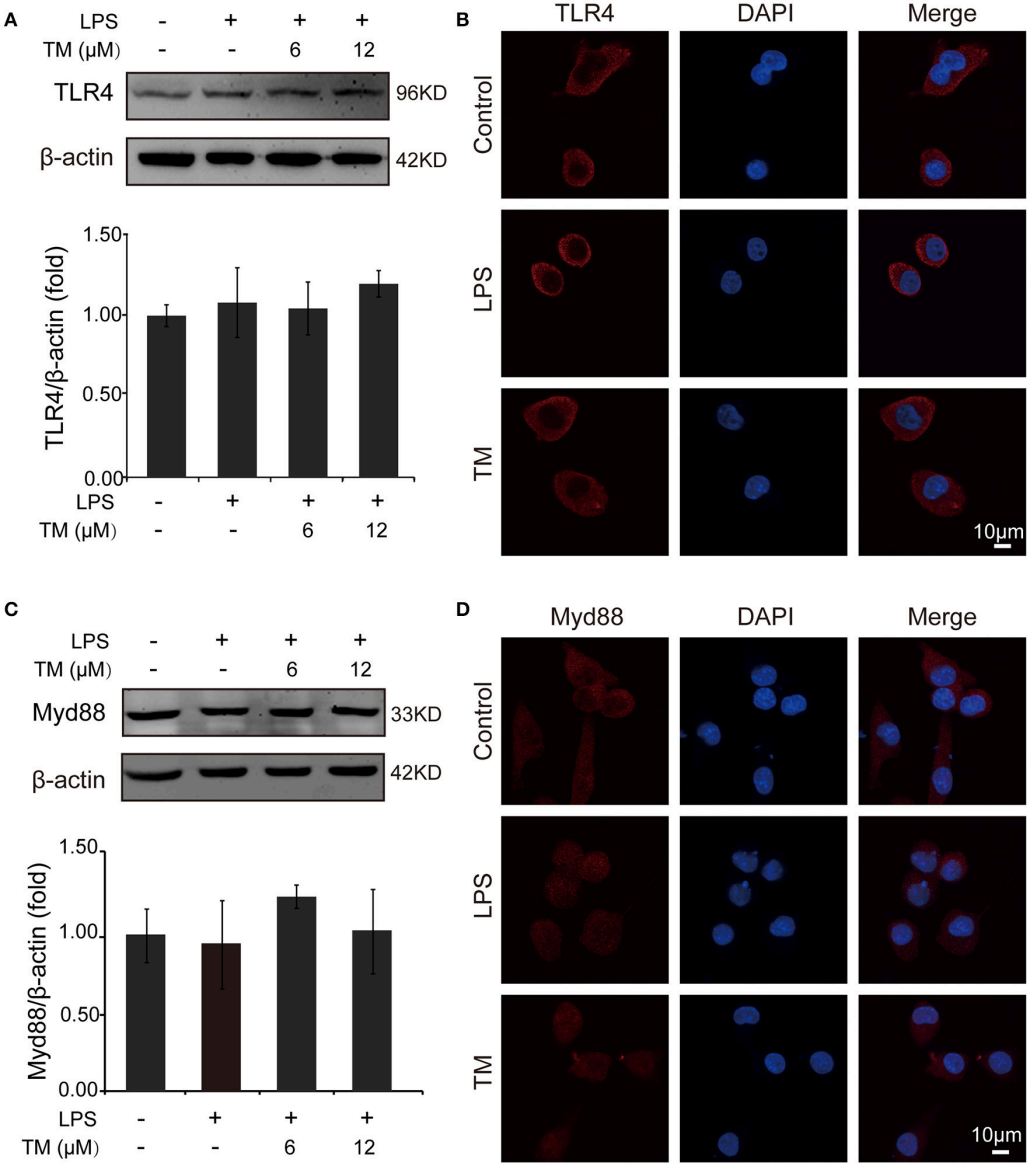
TM-pretreatment has no effect on TLR4 and MyD88 in BV-2 cells induced by LPS. BV-2 cells were pretreated with 6, 12 μM of TM, followed by treatment with 1 μg/ml LPS for 18 h. **(A,B)** The expression of TLR4 were determined by western blot (upper panel: representative pictures; lower panel: quantifications) and immunofluorescence. **(C,D)** The expression of Myd88 were determined by western blot (upper panel: representative pictures; lower panel: quantifications) and immunofluorescence. The results are expressed as the mean ± *SD*. of at least three independent experiments (*N* ≥ 3).

**Figure 5:**
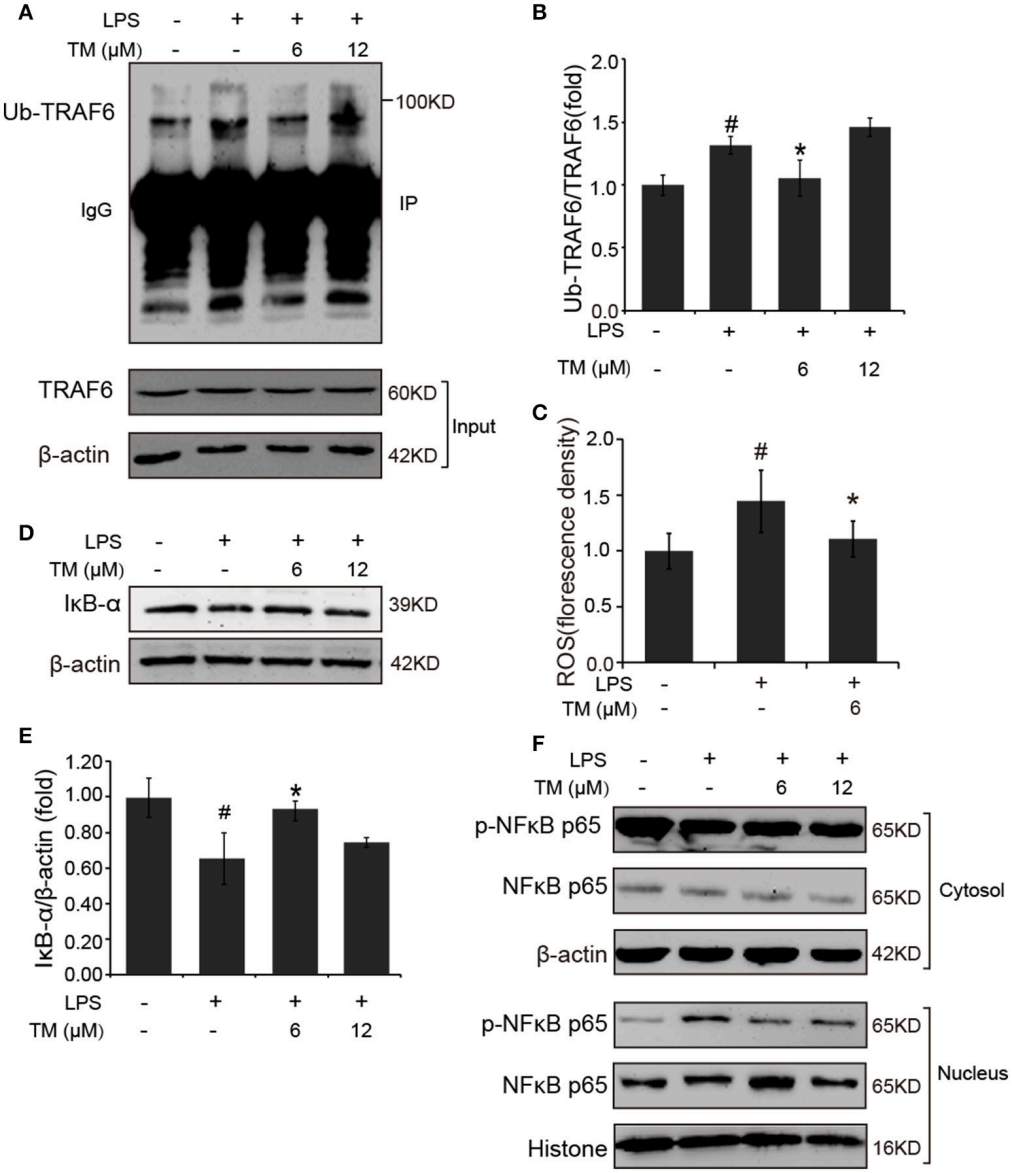
TM-pretreatment reduces the ubiquitination of TRAF6 and decreases nuclear translocation of NFκB by inhibiting the degradation of IκB-α in LPS-induced BV-2 cells. After treated with TM and LPS, cell lysates were collected and immunoprecipitated with TRAF6 antibody, and the IP sample was detected with ubiquitin antibody. **(A,B)** Immunoblot images **(A)** and quantifications **(B)** show that TM-pretreatment lead to the decrease of TRAF6 ubiquitination. **(C)** ROS was detected by fluorescent enzyme analyzer. **(D,E)** Immunoblot images **(D)** and quantifications **(E)** show that IκB-α expression was inhibited by LPS, but the inhibition was blocked by pre-treatment with TM. **(F)** Immunoblot images show that TM prohibited the nucleus localization of NFκB induced by LPS. The results are represented as the mean ± *SD* of at least three independent experiments (N ≥ 3). ^*^p < 0.05 compared with the LPS; #*p* < 0.05 compared with the control. The *p-*values were calculated by One-way ANOVA followed by Bonferroni's *post-hoc* test.

TLR4 signaling pathway plays a pivotal role in the inflammatory response. Studies have shown that TLR4/Myd88/NFκB signaling pathway is involved in LPS-stimulated BV-2 cells (Dai et al., 2015). On the basis of the above results that the effects of TM on LPS-stimulated BV-2 cells have no dose dependence, 6 μM TM and 12 μM TM were used for the following studies. First, We examined the effect of 6 μM and 12 μM TM on the expression of TLR4 and Myd88 in LPS-induced BV-2 cells, and observed that TM had no effect on the expression of TLR4 (Figures 4A,B, *p* > 0.05) and Myd88 (Figures 4C,D, *p* > 0.05). In addition, we also examined the effects of TM on the expression of TLR4 and Myd88 in APP/PS1 Tg mouse brains. Consistent with the results of the cell model, no changes were observed on the expression of TLR4 and Myd88 in APP/PS1 Tg mouse brains (Supplementary Figure 4), suggesting that TM regulated the inflammatory process in BV-2 cells without affecting the expression of the two key molecules, which mediated LPS-induced inflammation. It has been suggested that iron generates superoxide anion to induce ubiquitination of TRAF6 (Zhong et al., 2012). Copper, like iron, is a redox metal that produces free radicals (Greenough et al., 2013). In light of these results, we speculated that TM may reduce the ROS production and then regulate the activity of downstream signaling molecules in TLR4 signaling pathway, such as the ubiquitination of TRAF6, the phosphorylation of AKT and NFκB, and finally reduce the secretion of inflammatory cytokines. To confirm whether TM can reduce the production of ROS to reduce the ubiquitination of TRAF6, we detected the ubiquitination of TRAF6 by using the immunoprecipitation, and found that the LPS-induced ubiquitination of TRAF6 can be abolished by only pre-treated with 6 μM TM for 30 min (Figures 5A,B). Next, we detected the production of ROS induced by LPS with or without TM-treatment. The results showed that the production of ROS induced by LPS could be decreased by pre-treated with 6 μM for 30 min (Figure 5C). As IκB-α is the major negative regulator and the binding partner of NFκB, we determined the expression of IκB-α in the presence or absence of TM in LPS-induced BV-2 cells, and found that the expression of IκB-α was significantly induced in 6 μM TM-pretreated group, as compared with the LPS-induced group (Figures 5D,E, *p* < 0.05). To evaluate the nucleus translocation of phosphor-NFκB p65 subunit (p-NFκB p65) in LPS-induced BV-2 cells with or without TM-pretreatment, we isolated the cytoplasmic and the nucleus fraction of the treated cells, and detected the expression of p-NFκB p65 and NFκB p65 by western blot. The results indicated that TM-pretreatment significantly suppressed the nucleus translocation of p-NFκB p65 in LPS-induced BV-2 cells (Figure 5F), suggesting that TM treatment reduced inflammatory effects of LPS in BV-2 cells by down-regulating TRAF6/NFκB signaling pathway.

**Figure 6:**
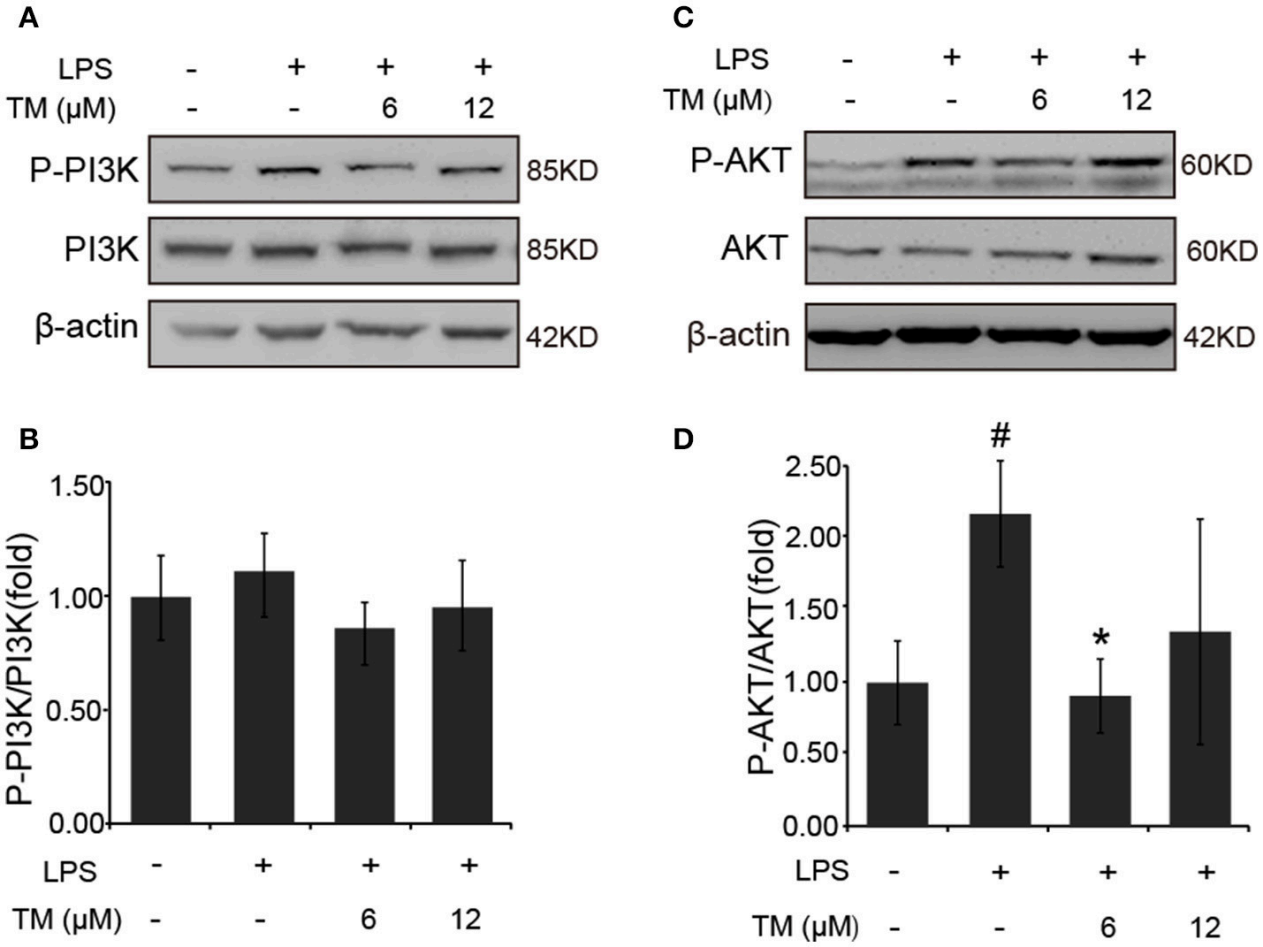
TM-pretreatment significantly decreases AKT phosphorylation in LPS-induced BV-2 cells. **(A,B)** Immunoblot images **(A)** and quantifications **(B)** show that both TM-pretreatment and LPS-stimulation did not affect PI3K expression in BV2 cells. **(C,D)** Immunoblot images **(C)** and quantifications **(D)** show that TM-pretreatment lead to a reduction of AKT phosphorylation induced by LPS. The results are represented as the mean ± *SD* of at least three independent experiments (*N* ≥ 3). ^*^p < 0.05 compared with the LPS; #*p* < 0.05 compared with the control. The *p-*values were calculated by One-way ANOVA followed by Bonferroni's *post-hoc* test.

It was previously reported that the PI3K/AKT pathway, the upstream activator of NFκB, was involved in the LPS-induced inflammatory responses (Venkatesan et al., 2010; Saponaro et al., 2012). Therefore, we examined the effect of TM on the activation of PI3K and AKT in LPS-induced BV-2 cells. Since, AKT activation requires the phosphorylation of Thr308 in the activation loop and Ser473 within the carboxyl-terminal hydrophobic motif. We detected Ser473 phosphorylation of AKT in LPS-induced BV-2 cells in the presence or absence of TM-pretreatment. Although no changes were detected in the phosphorylation of PI3K and total AKT in TM-treated APP/PS1 Tg mice (Supplementary Figure 5), lower dose of TM-pretreatment significantly decreased the Ser473 phosphorylation of AKT (Figures 6C,D, *p* < 0.05) induced by LPS in BV-2 cells without affecting PI3K activity (Figures 6A,B), suggesting that TM suppressed the production of inflammatory cytokines via reducing NFκB-nucleus localization which was mediated by TRAF6 auto-ubiquitination and AKT activation.

## Discussion

Neuroinflammation has a vital impact on the pathology of AD (Choo et al., 2013; Ardestani et al., 2017; He et al., 2017). Previous study showed that proinflammatory cytokines, such as IL-6, IL-1β, and TNF-α, were increased in the brain of APP/PS1 Tg mice (Song et al., 2014). Abnormal upregulation of various cytokines and chemokines exert proinflammatory response in AD brains (Flanders et al., 1995; Grammas and Ovase, 2001; Lue et al., 2001). Copper, one of redox-active transition metal ions, is enriched in brains of AD patients (Choo et al., 2013) and plays an important role in inflammation-associated diseases (Jomova et al., 2010). It can induce IL-6 secretion (Schmalz et al., 1998) and elevate neuroinflammatory responses (Kitazawa et al., 2016). Here we showed that TM, a copper chelator, could reduce intracellular bioavailable copper, ROS production and then suppressed the activity of TRAF6/NFκB signaling pathway and the secretion of inflammatory cytokines.

Microglia is the key innate immune cell that mediates inflammatory process of AD brains (Choo et al., 2013). Once activated, microglia secretes a variety of proinflammatory factors that are believed to exacerbate neurodegeneration (Liu and Hong, 2003). Hence, we utilized LPS-stimulated BV-2 cells as inflammatory cell model and found that TM abolished the activation of NFκB and the production of proinflammatory cytokines induced by LPS. Consistent with previous studies (Askari et al., 2004; Song et al., 2008; Wei et al., 2011), TM significantly decreased the production of inflammatory cytokines, such as TNF-α, IL-1β, and NO, in LPS-stimulated BV-2 cells. Meanwhile, it also reduced the mRNA and the protein expression of iNOS in BV-2 cells induced by LPS. These results indicated that TM treatment led to a decrease in the production of inflammatory cytokine via the transcriptional suppression. Furthermore, TM treatment led to the suppression of iNOS and TNF-α in APP/PS1 Tg mice. Although we did not observe any differences in other inflammatory cytokines, we considered that TM might reduce inflammatory responses by scavenging or degradation inflammatory factors *in vivo*.

TLR4 isone of cell surface receptors expressed on microglia that plays a pivotal role in initiating immune responses and regulates neuroinflammatory diseases (Lehnardt et al., 2003; Wang et al., 2009). It has been reported that TLR4/Myd88/NFκB signaling pathway is involved in the production of proinflammatory cytokines in LPS-induced BV-2 cells (Dai et al., 2015). TLR4 signaling pathways can elicit the activation of NFκB by Myd88 dependent pathway (Youn et al., 2006). Once stimulated by LPS, TLR4 recruits Myd88 protein to the cell membrane, then promotes the ubiquitination of TRAF6 (Barton and Medzhitov, 2003). The ubiquitinated-TRAF6 will activate TAK1/IKK/NFκB signaling pathway (Liu and Chen, 2011), and then induce the transcription of proinflammatory cytokines (Mankan et al., 2009; Zhang, L. et al., 2013). In addition to TLR4/Myd88 signaling pathway, other molecules, such as ${\text{O}}_{2}^{-}$, are also involved in the ubiquitination of TRAF6 (Zhong et al., 2012) and its downstream signaling pathways. In this study, we showed that TM abolished the nucleus localization of NFκB without affecting TLR4 and Myd88 expression *in vivo* or *in vitro*. As copper can produce reactive free radicals through Fenton reaction (Greenough et al., 2013), the levels of copper in the cells increased significantly after LPS stimulation, which may lead to the production of ROS, including ${\text{O}}_{2}^{-}$, and promote the ubiquitination of TRAF6 for TAK1 activation. Combined with the previous studies, we may infer that reducing intracellular free copper will inhibit ${\text{O}}_{2}^{-}$ production, and elicit TRAF6-mediated activation of TAK1. Consistent with the previous study (Wei et al., 2014), our results indicated that TM reduced intracellular bioavailable copper through formation of complex with copper and its chaperon, inhibiting copper transport into cells. The decrease of copper level in cells will reduce the generation of oxygen free radicals, the ubiquitination of TRAF6 and finally reduce the transcription of inflammatory cytokines through attenuation of NFκB activation (Pan et al., 2002, 2003).

The superoxide radical is one of the most abundant ROS within the cells that affects synaptic plasticity (Tejada-Simon et al., 2005) and neuronal lose (Harman, 2002; Resende et al., 2008). It has been reported that SOD1 is one of the essential antioxidant enzymes and its activity is reduced in AD patients (Marcus et al., 1998). The deficiency SOD1 accelerated Aβ oligomerization and memory impairment in Tg2576 mouse model of AD (Murakami et al., 2011), suggesting that SOD1 may work as a protector in neurodegenerative diseases. The activity of SOD1 requires the binding of copper and zinc ions(Fukuoka et al., 2017) and the copper chaperone for superoxide dismutase (CCS), which is required for copper incorporation into the SOD1 (Wong et al., 2000). Previous study showed that TM treatment led to a decrease in free copper (Zhang et al., 2015). In our research, TM has no effect on copper concentrations in APP/PS1 mouse brains; however TM treatment leads to an increase in SOD1 activation via CCS. In addition, TM acts as an anti-inflammatory agent, and inhibits LPS-induced inflammatory responses *in vivo* (Wei et al., 2011). The inhibitory effect of TM on TNF-α-induced NFκB activation was associated with the dephosphorylation and the degradation of IκBα (Wei et al., 2014). In our study, we showed that TM could attenuate neuroinflammation by decreasing the levels of TNF-α and IL-1β in microglial cells, whereas the secretion of these inflammatory cytokines might be associated with SOD1 activity and copper bioavailability.

Moreover, the ubiquitination process mediated by TRAF6 is required for the activation of TAK and AKT (Xia et al., 2009; Yang et al., 2009). Our data indicated that LPS induced TRAF6 auto-ubiquitination, resulting in AKT activation *in vitro*. TM treatment reduced the production of ROS, suppressed TRAF6 auto-ubiquitination and then regulated NFκB activation. Interestingly, we found that only p-NFκB p65 was reduced in the nuclear, whereas, the nuclear-translocation of NFκB p65 was not affected by TM-treatment. Our results suggested that TM suppressed the degradation of IκB-α, the nuclear-translocation of p-NFκB p65 and then down-regulated the transcription of proinflammatory cytokines.

**Figure 7:**
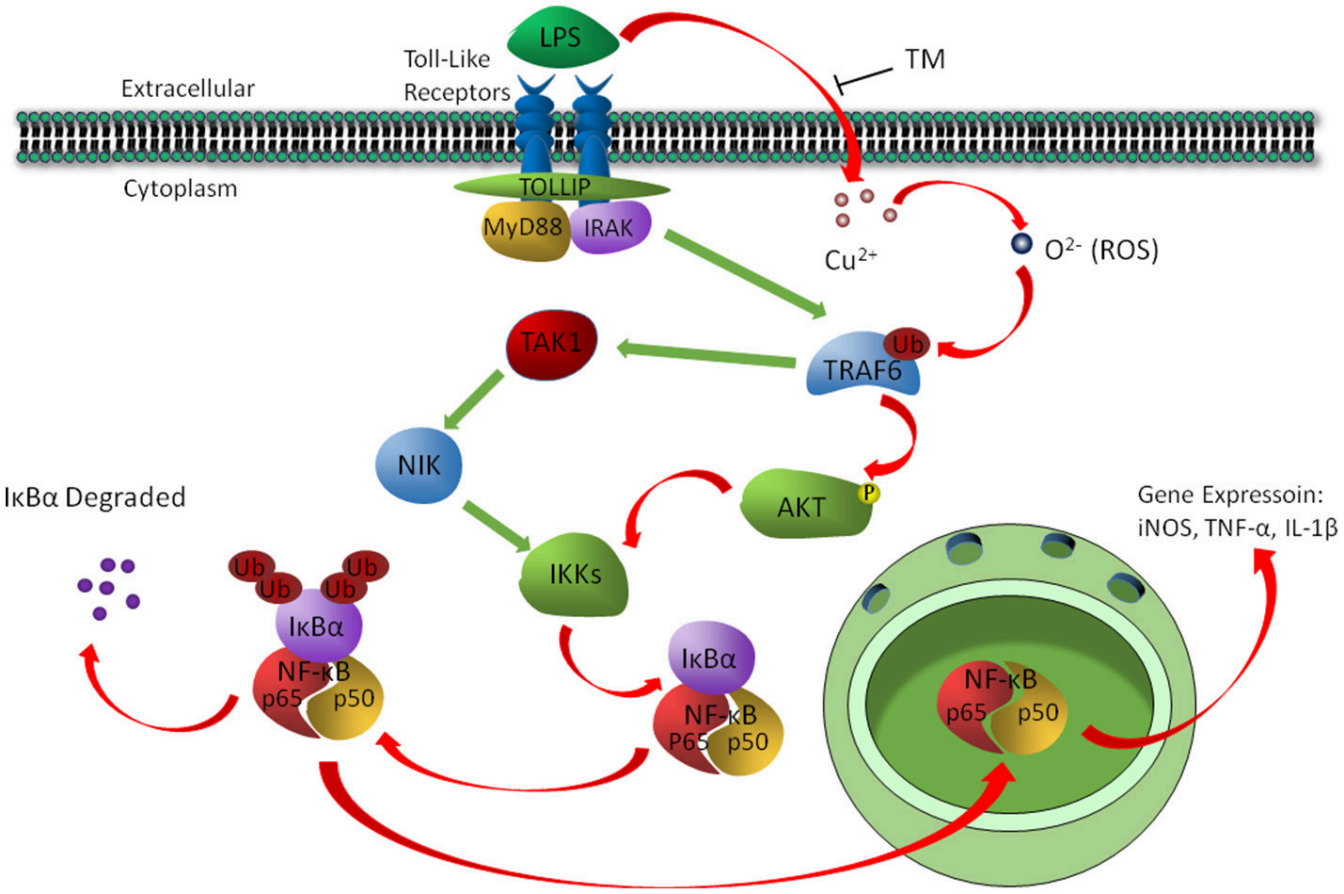
The schematic diagram shows that TM suppressed the secretion of NO, TNF-α, and IL-1β by reducing the bioavailability of copper and the activity of its downstream ROS/TRAF6/AKT/NFκB signaling pathway.

In conclusion, the present study revealed a signaling pathway by which TM treatment suppressed the inflammatory responses in LPS-induced BV-2 cells. We found that TM suppressed the secretion of NO, TNF-α and IL-1β *in vitro* by reducing the bioavailability of copper and the activity of its downstream ROS/TRAF6/AKT/NFκB signaling pathway, which resulted in the suppression of inflammatory responses in BV-2 cells (Figure 7).

## Author contributions

Z-YW and PZ designed the study. ZW with the help of Y-HZ, CG, H-LG, M-LZ, T-TH, N-NL, and R-FG performed most of the experiments. TL and WZ assisted with immunofluorescence experiments. ZW, Z-YW, and PZ wrote the paper.

### Conflict of interest statement

The authors declare that the research was conducted in the absence of any commercial or financial relationships that could be construed as a potential conflict of interest.
