# Supplementary material for: Tetrathiomolybdate Treatment Leads to the Suppression of Inflammatory Responses through the TRAF6/NFκB Pathway in LPS-Stimulated BV-2 Microglia
Source: Front Aging Neurosci. 2018 Feb 27;10:9. doi: 10.3389/fnagi.2018.00009 (PMC5835334; doi:10.3389/fnagi.2018.00009)
Supplement: Supplementary file 1 [file DataSheet1.DOCX]

Supplementary Material

Tetrathiomolybdate treatment lead to suppress inflammatory responses through the TRAF6/NFκB pathway in LPS-stimulated BV-2 microglia

Zhuo Wang ^a^, Ya-Hong Zhang ^a^, Chuang Guo ^a^, Hui-Ling Gao ^a^, Ting-Ting Huang ^a^ Na-Na Liu ^a^, Rui-Fang Guo ^a^, Tian Lan ^a^,Wei Zhang ^b^, Zhan-You Wang ^a,^*, and Pu Zhao ^a,^*

*** Correspondence:** zhaopu6687700@mail.neu.edu.cn (Pu Zhao); wangzy@mail.neu.edu.cn (Zhan-You Wang)

**Supplementary Figures**


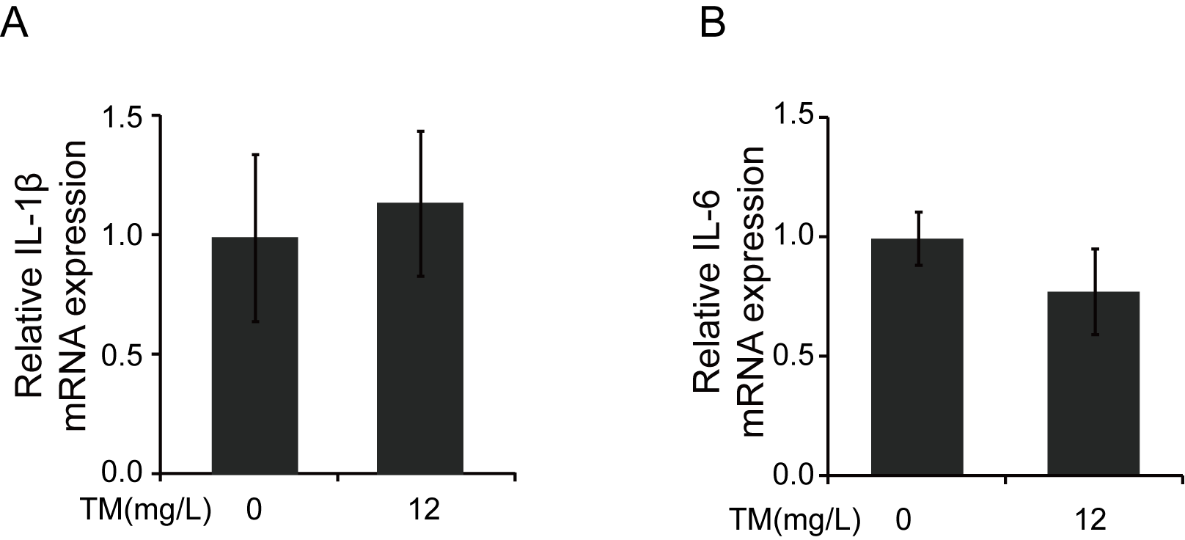


Supplementary Figure 1. APP/PS1 Tg mice were pretreated with TM for 3 months and the brain homogenates were used to analyze the effects of TM on the mRNA expression of IL-1β and IL-6. (A-B) Quantitative real-time PCR results show that TM-treatment has no effect on the mRNA expression of IL-1β and IL-6. Data were represented as means ± S.D.. N = 6 mice per group. **p* < 0.05, ***p* < 0.01. The *p* values were calculated using 2-tailed Student’s *t*-test.


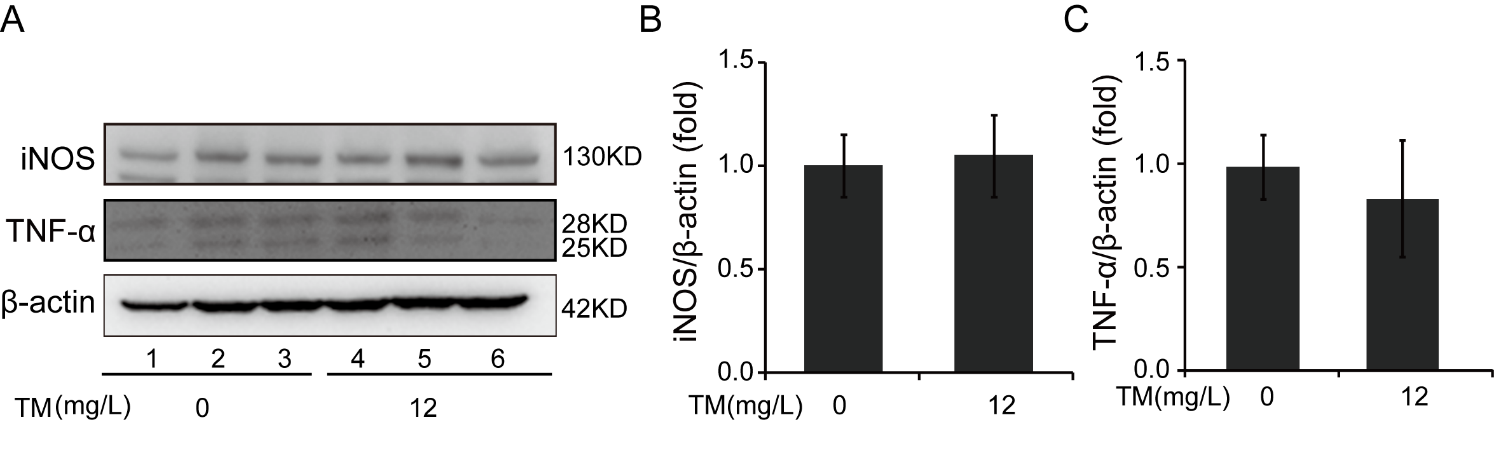


Supplementary Figure 2. Wild-type (WT) mice were pretreated with TM for 3 months and the brain homogenates were used to analyze the effects of TM on the expression of iNOS and TNF-α in WT mice. (A-C) Immunoblot images (A) and quantifications (B ,C) show that TM-treatment has no effect on the expression of TNF-α (A,B) and the expression of iNOS (A,C). Lanes 1-3 represented different individuals of control WT mice. Lanes 4-6 represented different individuals of TM-treated WT mice. Data are represented as means ± S.D.. N = 6 mice per group. **p* < 0.05, ***p* < 0.01. The *p* values were calculated using 2-tailed Student’s *t*-test.

**
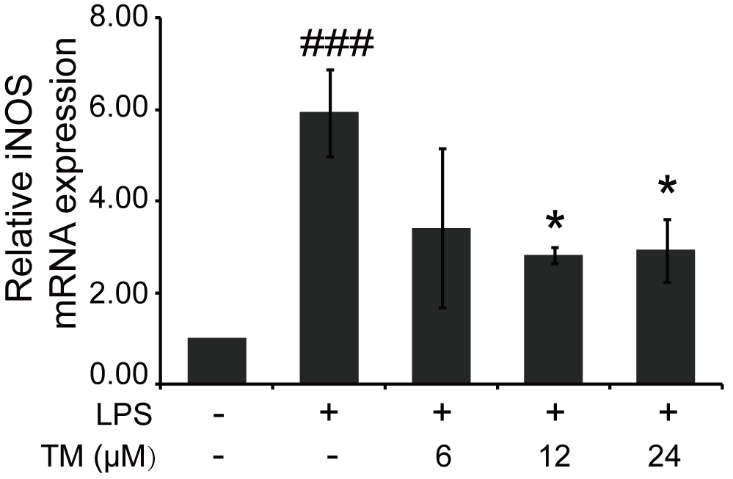
**

Supplementary Figure 3. Quantitative real-time PCR data show that TM blocks the mRNA expression of iNOS in LPS-induced BV2 cells. Data are represented as means ± S.D. of at least three independent experiments (N ≥ 3). * *p* < 0.05 compared with the LPS; ### *p* < 0.001 compared with the control. The *p* values were calculated by One-way ANOVA.


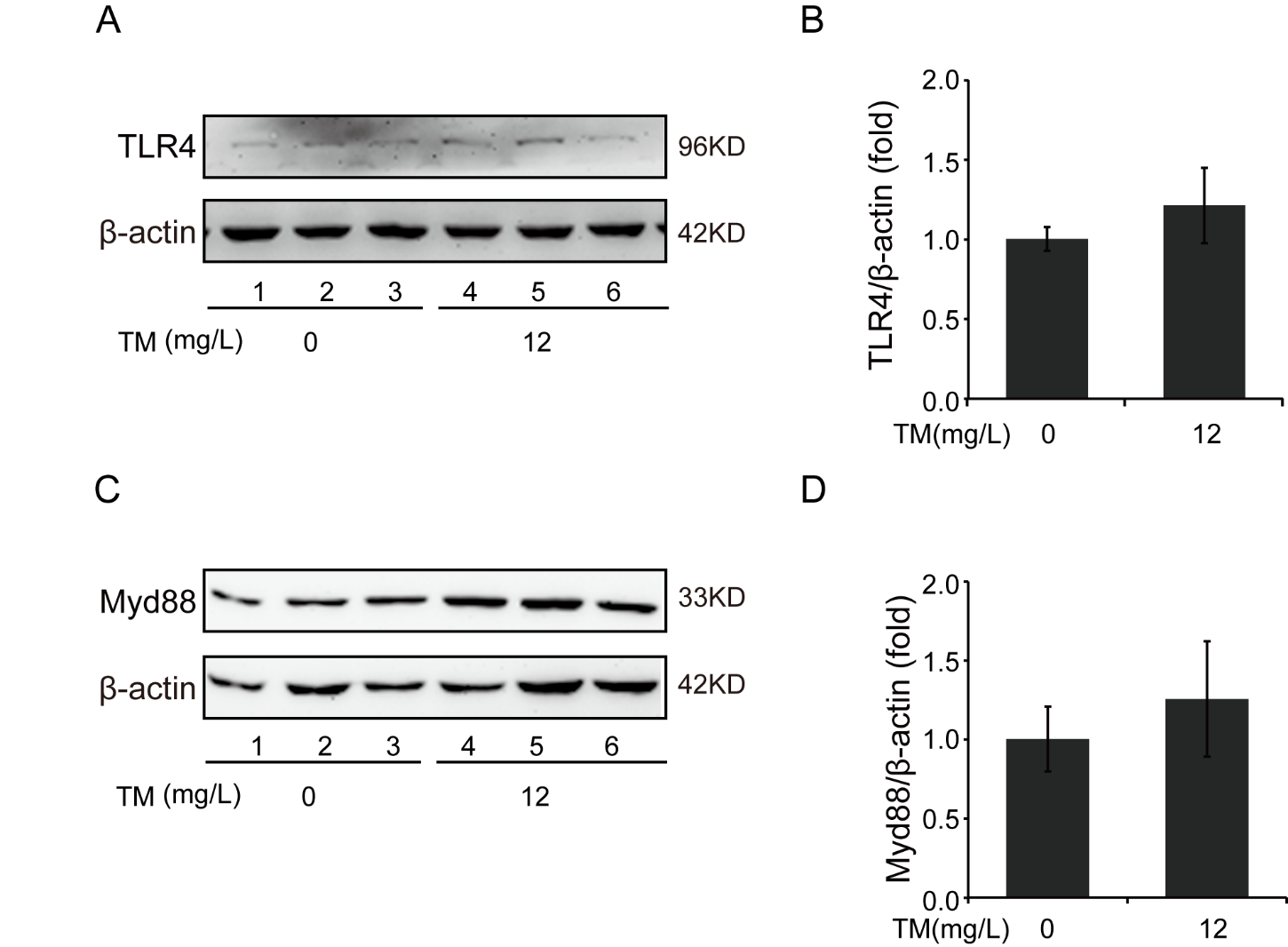
 Supplementary Figure 4. APP/PS1 Tg mice were pretreated with TM for 3 months and the brain homogenates were used to analyze the effects of TM on the expression of TLR4 and Myd88. (A-D) Immunoblot images (A,C) and quantifications (B,D) show the expression of TLR4 (A,B) and the expression of Myd88 in control and TM-treated APP/PS1 mice. Lanes 1-3 represented different individuals of control APP/PS1 mice. Lanes 4-6 represented different individuals of TM-treated APP/PS1 mice. Data are represented as means ± S.D.. N = 6 mice per group. **p* < 0.05, ***p* < 0.01. The *p* values were calculated using 2-tailed Student’s *t*-test.


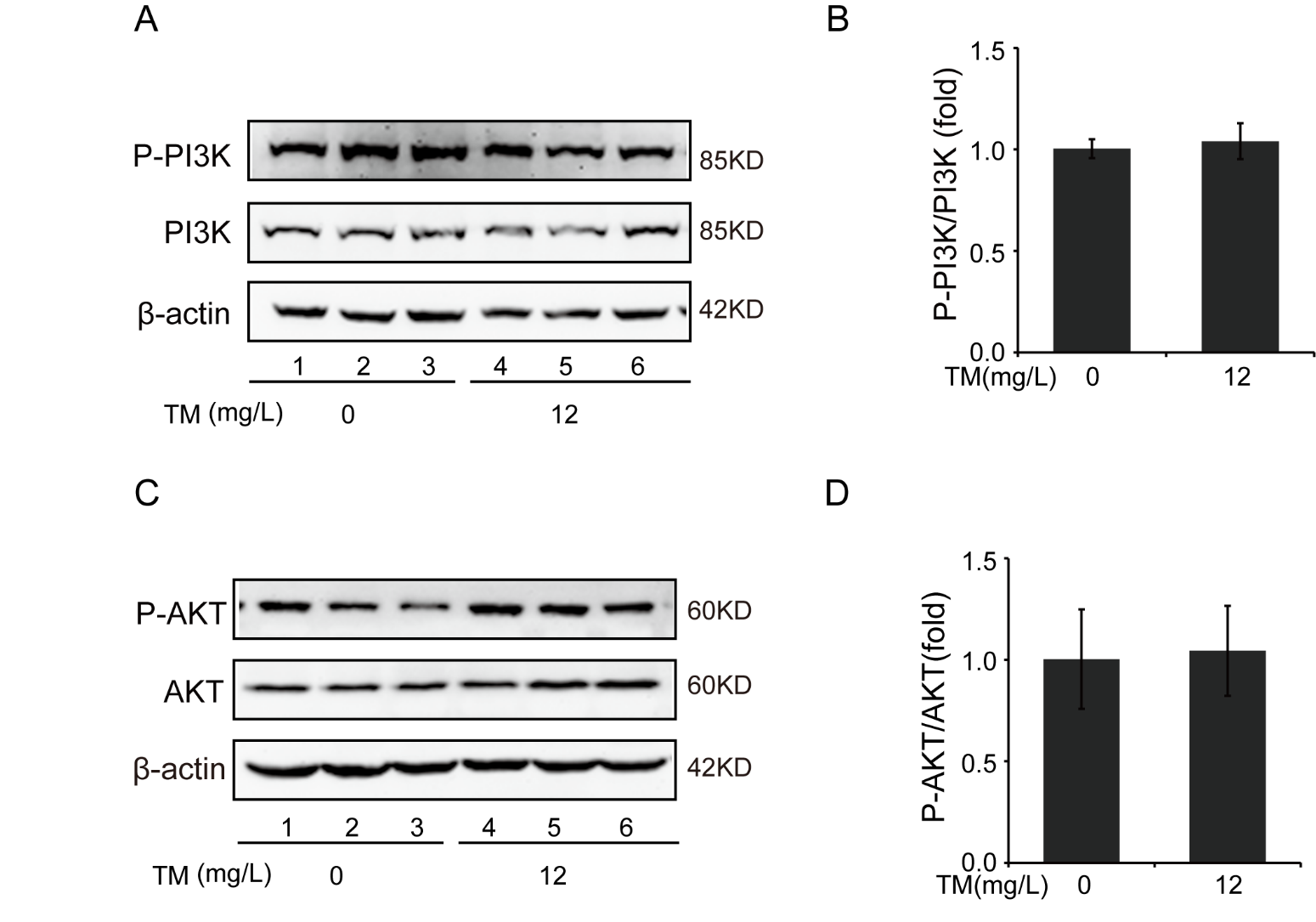


Supplementary Figure 5. APP/PS1 Tg mice were pretreated with TM for 3 months and the brain homogenates were used to analyze the effects of TM on the phosphorylation of PI3K and AKT. (A-D) Immunoblot images (A,C) and quantifications (B,D) show that TM-treatment has no effect on PI3K and AKT phosphorylation in APP/PS1 mice. Lanes 1-3 represented different individuals of control APP/PS1 mice. Lanes 4-6 represented different individuals of TM-treated APP/PS1 mice. Data are represented as means ± S.D.. N = 6 mice per group. **p* < 0.05, ***p* < 0.01. The *p* values were calculated using 2-tailed Student’s *t*-test.

**SUPPLEMENTAL TABLE 1. Antibody sources and conditions**

| Antibody | Host | Dilution WB | Source(reference) |
| --- | --- | --- | --- |
| TLR4 | Rabbit | 1:500 | Sangon, Shanghai, China (D220102) |
| MyD88 | Rabbit | 1:500 | Sangon, Shanghai, China (D121009) |
| p-AKT | Rabbit | 1:1000 | Sangon, Shanghai, China (D155022) |
| AKT | Rabbit | 1:1000 | Sangon, Shanghai, China (D120056) |
| p-PI3K | Rabbit | 1:1000 | Cell Signaling Technology (#4228) |
| PI3K | Rabbit | 1:1000 | Cell Signaling Technology (#4257) |
| IκB-α | Mouse | 1:1000 | Cell Signaling Technology (#4814) |
| p-NFκB | Rabbit | 1:1000 | Cell Signaling Technology (#3033) |
| NFκB | Rabbit | 1:1000 | Sigma-Aldrich (SAB4502615) |
| iNOS | Rabbit | 1:500 | Sangon, Shanghai, China (D262121) |
| Ubiquitin | Rabbit | 1:1000 | Cell Signaling Technology (#3933) |
| TRAF6 | Rabbit | 1:1000 | Santa Cruz Biotechnology (SC-7721) |
| Histone | Rabbit | 1:2000 | Sangon, Shanghai, China (D154202) |
| TNF-α | Rabbit | 1:500 | Sangon, Shanghai, China (D221347) |
| β-actin | Mouse | 1:10000 | Sigma-Aldrich (A1978) |

Raw data for figure 1
